# Supplementary material for: Extracellular vesicle fusion visualized by cryo-electron microscopy
Source: PNAS Nexus. 2022 Aug 16;1(4):pgac156. doi: 10.1093/pnasnexus/pgac156 (PMC9802263; doi:10.1093/pnasnexus/pgac156)
Supplement: pgac156_Supplemental_Files [file pgac156_supplemental_files.zip › PNASNEXUS-PNASNEXUS-2022-00350-T-s01.docx]

Supplementary Materials for

**Extracellular vesicle fusion visualized by cryo-EM**

Mattia I. Morandi ^1^, Petro Busko ^1^, Efrat Ozer-Partuk ^1^, Suman Khan ^1^, Giulia Zarfati ^1^, Yael Elbaz-Alon ^1^, Paula Abou Karam ^1^, Tina Napso Shogan ^2^, Lana Ginini ^2^, Ziv Gil ^2,3^, Neta Regev-Rudzki ^1,*^ & Ori Avinoam ^1,*^

* Corresponding author. Email: [neta.regev-rudzki@weizmann.ac.il](mailto:neta.regev-rudzki@weizmann.ac.il), [ori.avinoam@weizmann.ac.il](mailto:ori.avinoam@weizmann.ac.il)

**This PDF file includes:**

Figs. S1 to S6

Captions for Movies S1 to S2

**Other Supplementary Materials for this manuscript include the following:**

Movies S1 to S2

**SUPPLEMENTARY FIGURES**

**Figure S1. (A)** EVs isolated via ultracentrifugation (green) compared to density gradient fractions grouped by density: fractions 1 - 3 (yellow) corresponding to 5% OptiPrep, fractions 4 - 6 (blue) corresponding to 10% OptiPrep, fractions 7 - 9 (orange) corresponding to 20% OptiPrep, and fractions 10 - 11 (gray) corresponding to 40% OptiPrep. Fractions 7 – 9 display a similar size distribution and concentration of particles to EVs isolated by ultracentrifugation. **(B)** Comparison of NTA size distribution of isolated sample from cell culture supernatant (green) and naïve growth media (orange) after differential ultracentrifugation.

**Figure S2. (A)** Schematics of image analysis performed on cryo-EM images of EVs’ and LUVs’ luminal regions. Representative cryo-EM images of EV and LUV and representative luminal regions (120 x120 pixels) were selected directly for spatial distribution analysis or transformed into binary images for Fourier analysis presented in Fig. 1 D. Scale bar: 100 nm. **(B)** Representative radial profiles (open symbols) of luminal gray level intensity for EV (red) and LUV (gray), their respective Savitzky – Golay smoothed profiles (dotted line) and relative peak finding analysis (red whiskers). **(C)** Summary of peak-peak distance distribution of luminal radial profiles for EVs and LUVs. **(D)** Summary of radial profiles of spatial autocorrelation function calculated for EV (red) and LUV (gray) luminal regions. Data are presented as average (open symbol) and standard deviation (whiskers) obtained from n = 10 EVs and n=10 LUVs.

**Figure S3. (A)** FRET fusion assay results for labeled retroviruses (green), EVs (orange), and LUVs (gray) incubated with unlabeled LUVs at pH ranging from 7.4 to 5.0, showing that a significant increase of the donor intensity occurs only upon progressive acidification. Purple-bound and transparent boxes indicate data also presented in Fig. 2 C, obtained from the same experiment.  **(B)** FRET fusion assay for EVs isolated from pooled density gradient fractions and via ultracentrifugation only, incubated with LUVs at pH 5.0. Only fractions 7 – 9 show a similar membrane mixing compared to control EVs. Grey-bound and transparent box indicates control data also shown in Fig. 2 E, obtained from the same experiment. **(C)** FRET fusion assay for OVCAR-3 cell culture media and naïve growth media, after ultracentrifugation-based isolation protocol (see Materials and Methods), incubated with late endosomal LUVs at pH 7.4 (green) and 5.0 (orange), showing that a significant increase of the donor intensity occurs only for samples isolated from cell supernatant, but not from growth media. **(D)** Mean diameter of suspension of either LUVs only or EVs incubated with LUVs, at pH 7.4 (green) or pH 5.0 (orange).

**Figure S4. (A)** Representative NTA size distribution curve comparing non-treated EVs (green) and EVs treated with Proteinase K at 37°C for 45 min (orange). **(B)** FRET fusion assay comparing EVs either treated with proteinase inhibitor PMSF or non-treated, incubated with late endosomal-mimicking LUVs at pH 5.0. In all panels, late endosomal-mimicking LUVs were utilized. Grey-bound and transparent box indicates control data also shown in Fig. 2 E, obtained from the same experiment. **(C)** Representative cryo-EM images of untreated EVs (left) and EVs treated with Protease K (right) showing an overall intact morphology and minimal membrane alteration.

**Figure S5.** Representative NTA size distribution curves comparing non-treated EVs (orange) and EVs pre-acidified at pH 5.0 without incubation with LUVs for 45 min at 4 °C (gray).

**Figure S6. (A)** Representative Cryo-EM images of LUVs at pH 7.4 (top) and pH 5.0 (bottom). At pH 7.4 LUVs do not significantly interact and display only minor contact sites with no extended interface between bilayers. Upon acidification, no fusion intermediates are observed. Scale bars 100 nm. **(B)** Tomographic reconstruction of vesicle – vesicle interaction at pH 5.0 reveals an expanded pore intermediate, with the EV cargo leaking into the liposomal lumen. Scale bar 100 nm.

**Movie S1.**

Tomogram showing content mixing between three vesicles at pH 5.0, corresponding to Fig. 4 G. Tomogram was acquired at defocus –3.5 and denoised using NAD filter with k value 5 and 5 iterations. Scale bar: 100 nm.

**Movie S2.**

Tomogram showing an expanded pore between vesicles at pH 5.0, corresponding to Fig. S5 B. Tomogram was acquired at defocus –3.5 and denoised using NAD filter with k value 5 and 5 iterations. Scale bar: 50 nm.

**Figure S1**

**
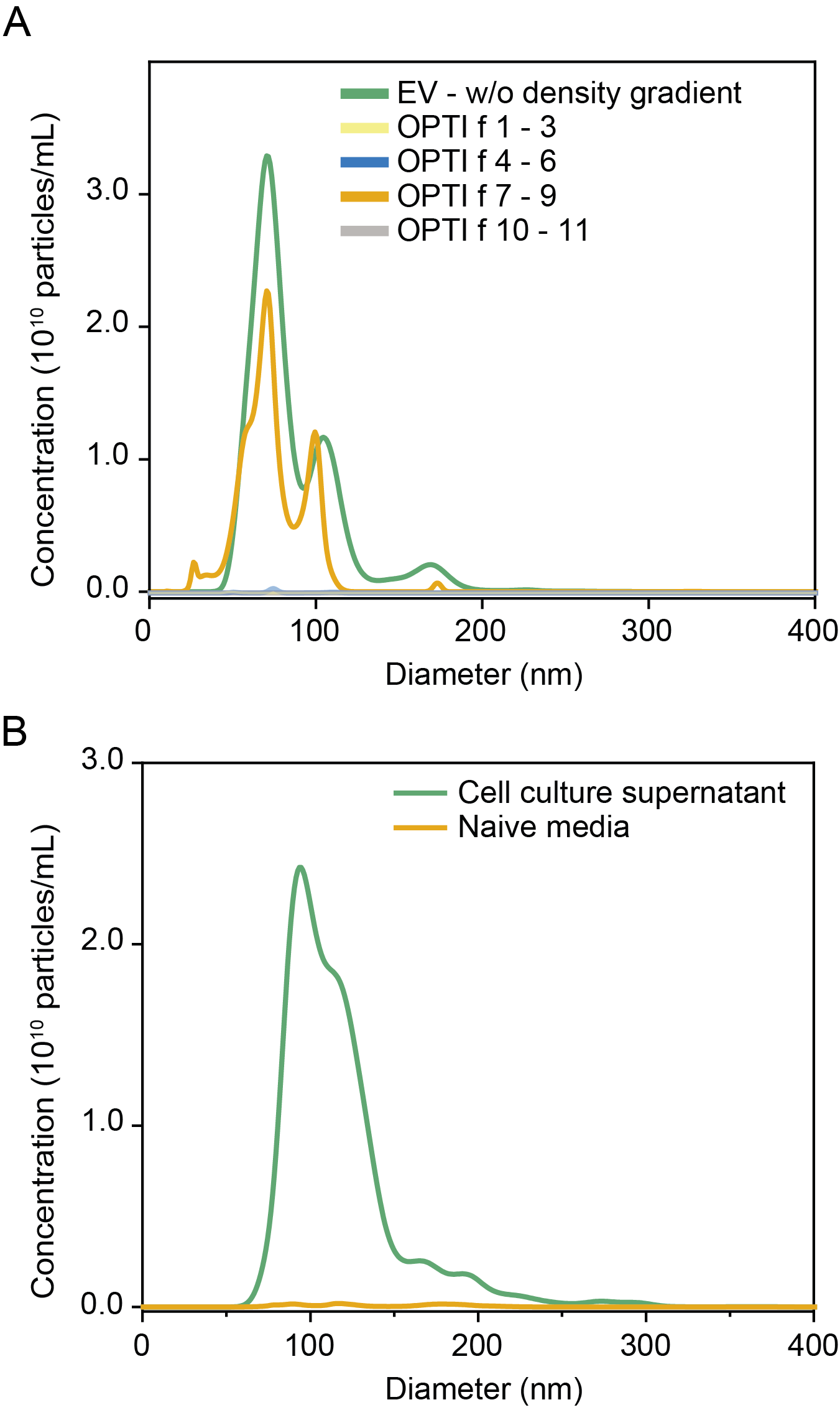
**

**Figure S2**

**
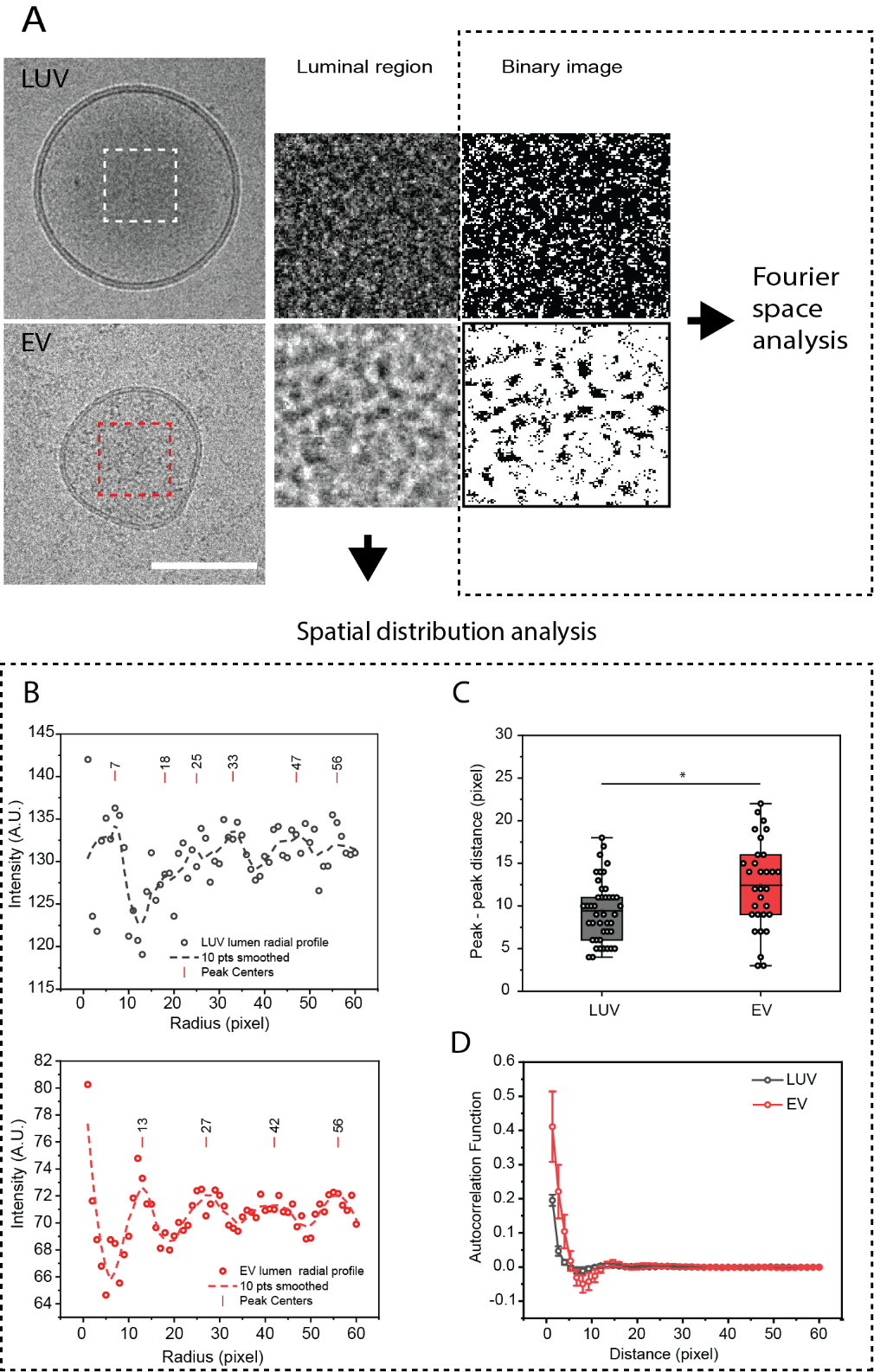
**

**Figure S3**


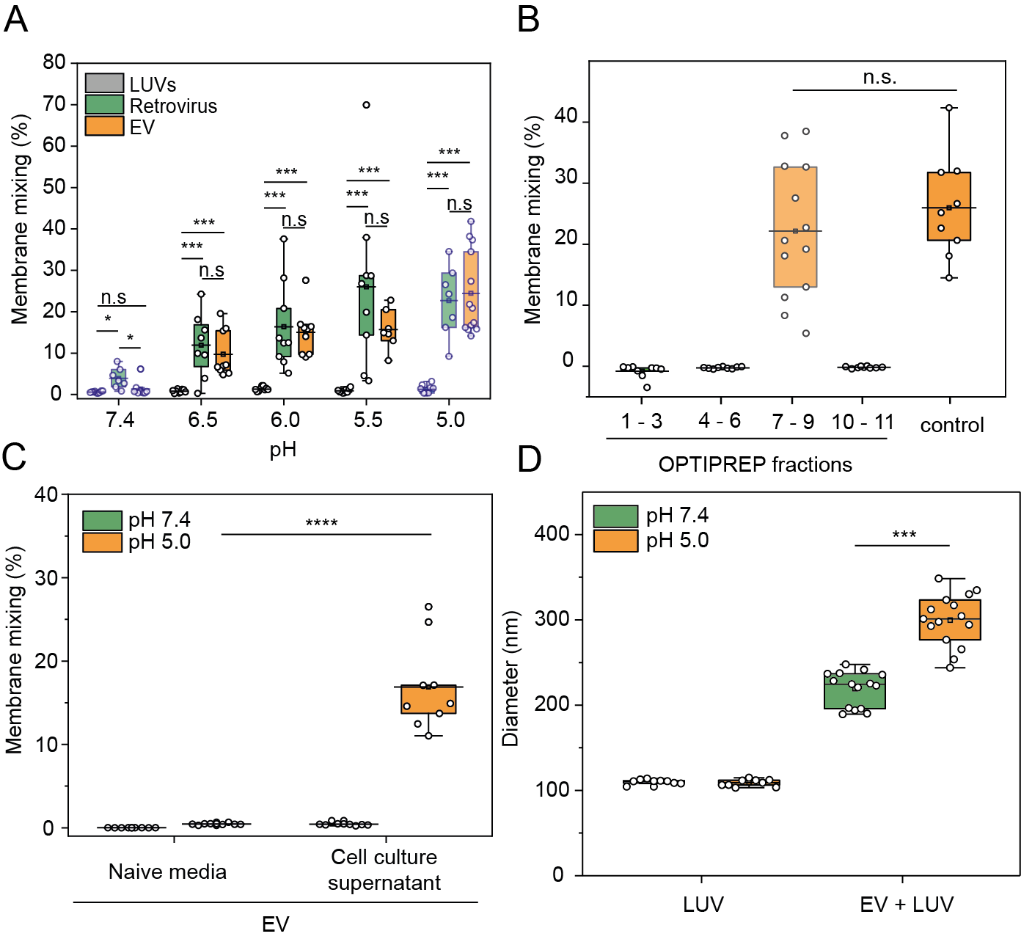


**Figure S4**

**
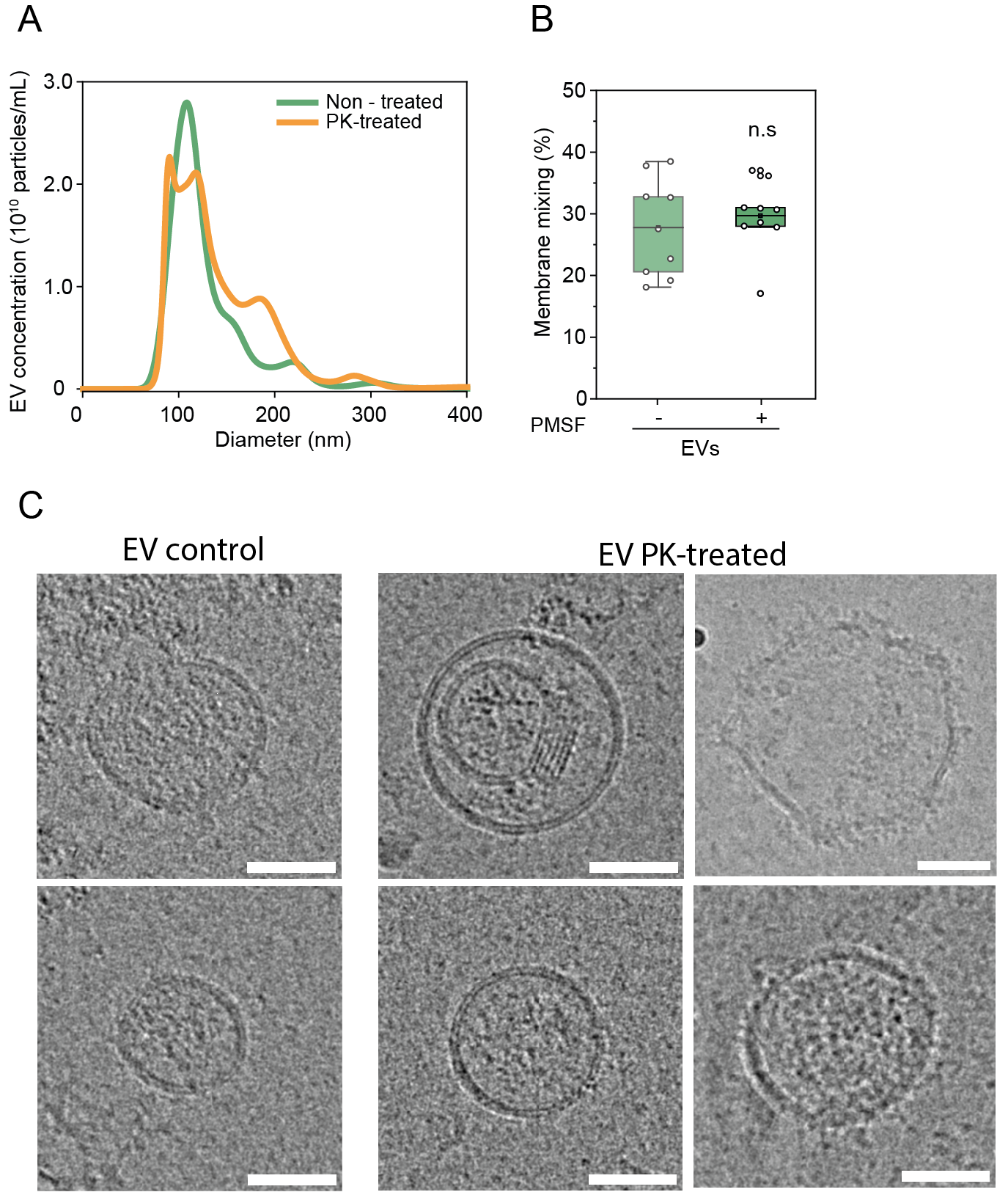
**

**Figure S5**

**
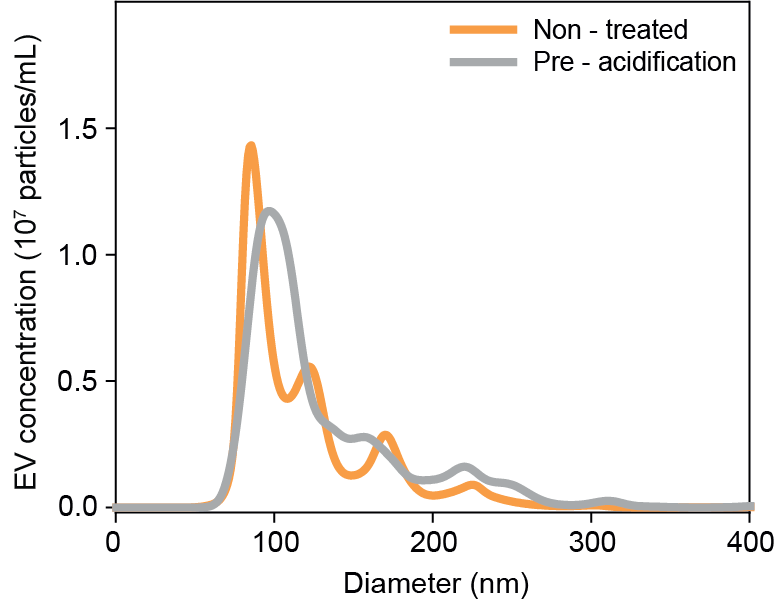
**

**Figure S6**

**
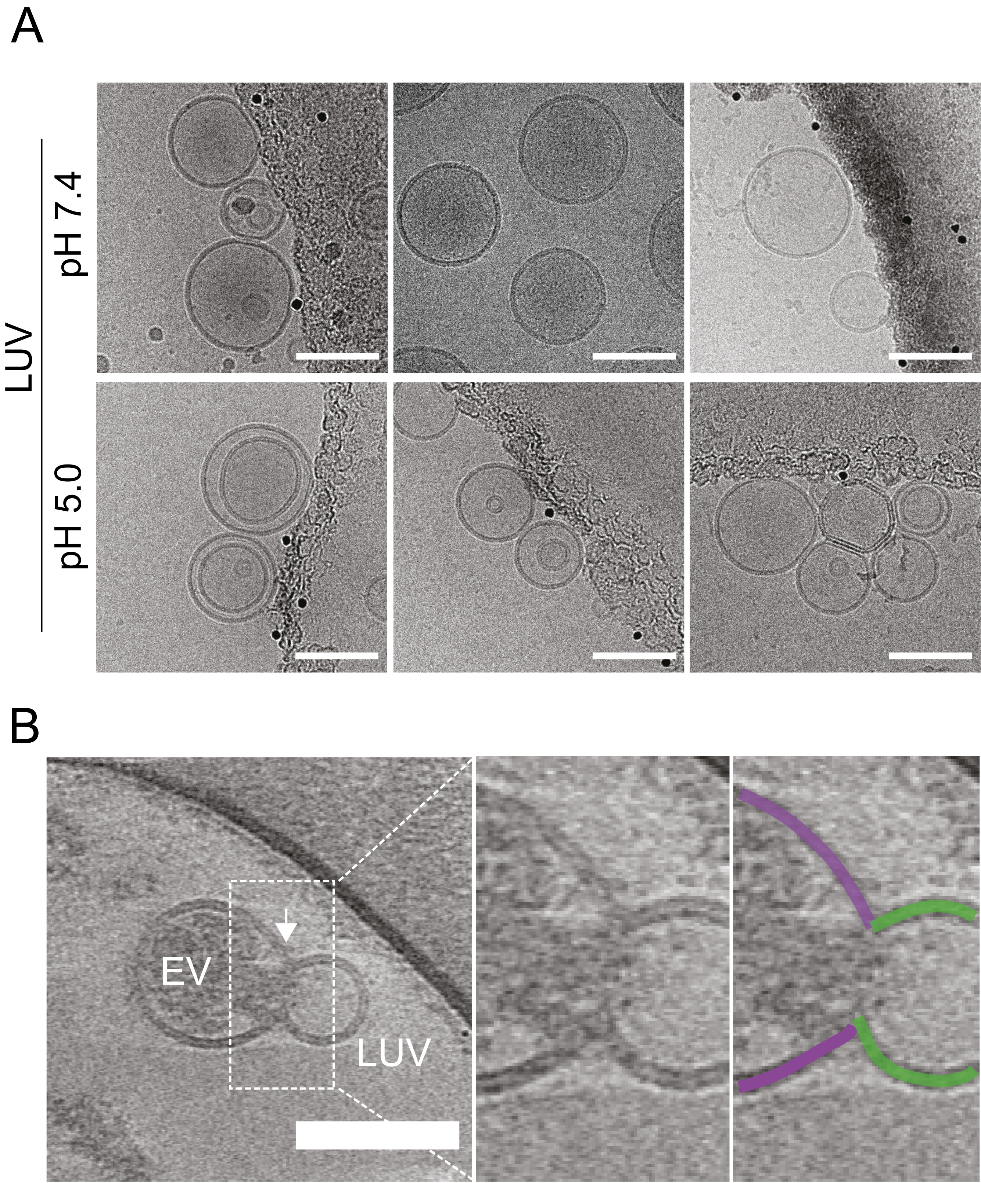
**
