# Supplementary figures and images for: Extracellular vesicle fusion visualized by cryo-electron microscopy
Source: PNAS Nexus. 2022 Aug 16;1(4):pgac156. doi: 10.1093/pnasnexus/pgac156 (PMC9802263; doi:10.1093/pnasnexus/pgac156)

Fig. 1

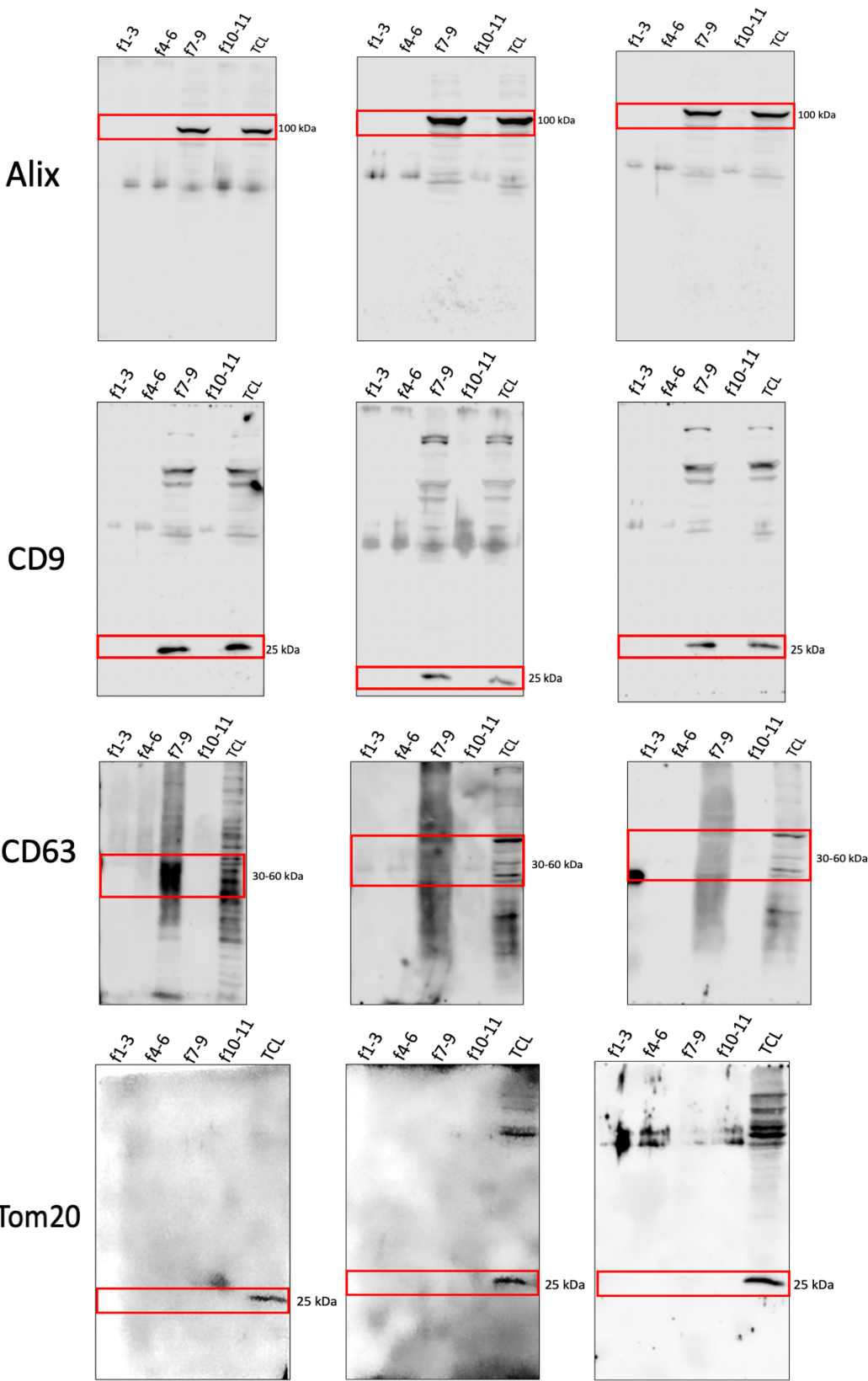

Fig. 3

Original membrane for Figure 3 C

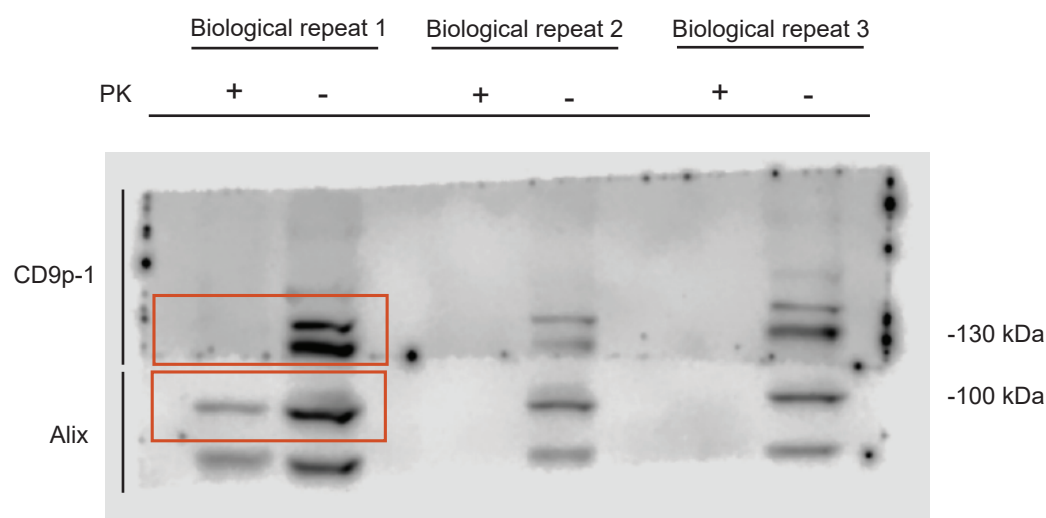

Supplement: pgac156_Supplemental_Files [file pgac156_supplemental_files.zip › PNASNEXUS-PNASNEXUS-2022-00350-T-s04.pdf]
